# Methodological foundations for locating the neural correlates of low-level visual experience

**DOI:** 10.3389/fpsyg.2026.1758591

**Published:** 2026-05-08

**Authors:** Benjamin Kozuch, Peter Kok

**Affiliations:** 1 Department of Philosophy, University of Alabama, Tuscaloosa, AL, United States; 2 Department of Imaging Neuroscience, UCL Queen Square Institute of Neurology, University College London, London, United Kingdom

**Keywords:** color, consciousness, methodology, neural correlates of consciousness (NCC), sensation

## Abstract

This article presents a framework for identifying the neural correlates of a low-level component of visual consciousness, *base experience*, defined as the spatial arrangement of colors experienced across the visual field. We propose a content-matching method that compares the content of base experience with that represented by candidate neural systems, treating content matches and mismatches as evidence for or against a neural system being the neural basis of base experience. To support this method, we introduce a formal system that specifies the content of both base experience and neural activity in a common quantitative format, allowing them to be precisely compared. We then outline three matching strategies—region-matching, grain-matching, and representational-matching—and show how each can be used to test hypotheses about the neural basis of conscious visual content. Finally, we consider how applying this method can help adjudicate between competing theories of consciousness.

## 1 Introduction

If anything qualifies as the holy grail of neuroscientific research into consciousness, it is identifying the neural correlates of consciousness (NCC). A complete account of the neural basis of consciousness might not resolve the hard problem, but would nonetheless mark a major advance in our understanding of one of the last great scientific and philosophical mysteries. Encouragingly, NCC research has made notable progress in recent years. Still, such progress should not deter us from exploring new approaches to NCC research, ones that might yield significant or rapid gains.

In this article, we present a theoretical framework for identifying the neural basis of a fundamental aspect of visual experience: *base experience*, understood as the spatial arrangement of colors that fills the conscious visual field. The method we propose is a form of content-matching, which seeks to identify the neural basis of an experience by assessing whether its content matches the content carried by a neural system (Noë and Thompson, 2004; Kozuch, 2015). We will show below that, because colors in visual experience are represented as being located in specific regions of the visual field, base experience has a distinctive spatial structure that lends itself to an especially fine-grained method for determining whether a neural system could be the neural basis of color experience. Because the content-matching method offered here requires substantial theoretical groundwork, our focus will remain on developing its theoretical foundations, with practical considerations addressed only briefly.

Here’s the article’s layout: In Section 1, we clarify how the idea of base experience should be understood; Section 2 introduces the content-matching method and how it can be used in NCC research; Section 3 presents a formal system for specifying the content of base experience; Section 4 presents a corresponding system for specifying the content of neural systems; Section 5 uses these systems to outline and discuss three ways of testing hypotheses about the neural basis of base experience; Section 6 summarizes the framework, notes its limitations, and outlines its promise for future NCC research.

## 2 Base experience

The NCC research method that we lay out below targets a specific type of conscious visual content: the spatial arrangement of colors represented across the conscious visual field. Because this arrangement appears to pervade visual experience, with experiences of other properties such as shape and motion seemingly inextricably linked to it, we refer to it as *base experience* (cf. Pollen, 2007). Since our aim is to identify the neural basis of this content, our focus is on what Chalmers (2000) calls a *content NCC*: the neural basis of what is represented in experience, such as color, motion, and shape (see also Kozuch and Kriegel, 2015). Accordingly, we largely set aside questions concerning the enabling NCC (Kozuch, 2024), understood as the neural systems that make the conscious experience of any content possible at all, prominent candidates here being the prefrontal cortex or a broader frontoparietal workspace.

### 2.1 What is base experience?

To better grasp what is meant by *base experience*, reflect on the character of your visual experience as it is right now. What you will notice is that, within your visual experience, there is a spatial arrangement of colors represented across the visual field. For example, when you look at a yellow banana on a white plate, yellow is represented in the region of the visual field corresponding to the banana, white is represented in the region corresponding to the plate, and other regions are filled with the colors of the remaining visible surfaces.

An instance of a base experience can be broken down into two components: (a) what colors one is experiencing in the visual field, and (b) where in the visual field those colors are represented. Let us look at demonstrations.

**Figure 1:**
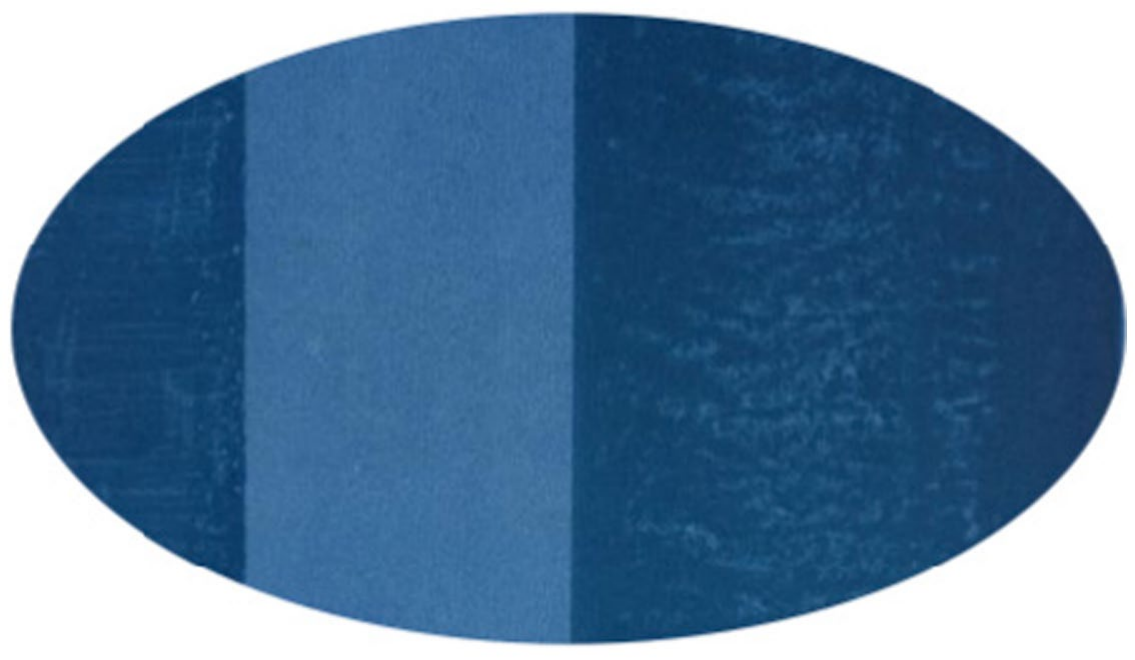
Simpler demonstration of base experience: See body for description.

**Figure 2:**
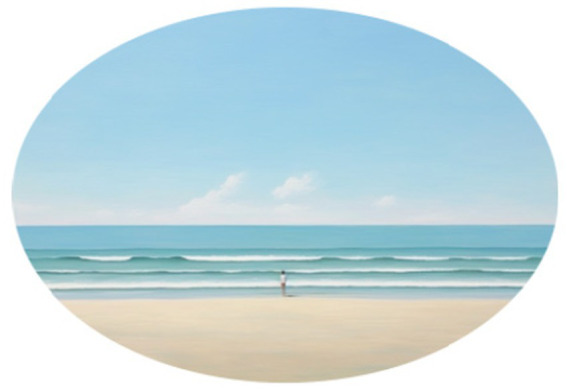
More complex demonstration of base experience: See body for description.

If the expanse of blue depicted in Figure 1 is what was in your conscious visual field, your base experience would consist of a representation of an expanse of dark blue throughout the entire visual field, except for the mid-left part of it, where a part of the visual field in the shape of a wide vertical stripe is represented as containing a lighter blue. Here’s another, slightly more complex demonstration:

If this beach scene in Figure 2 is what your visual experience was like, your base experience would consist of representations of:

- the tan of the sand in the lower part of the visual field.
- the blue-green of the water in the bottom middle part of the visual field, except where interrupted by the white of the breaking waves.
- the blue of the sky in the top part of the visual field, except where interrupted by the white of the clouds.

As we have seen, base experience is grounded in our experiences of color. However, color experience is often thought to decompose into multiple subcomponents, and what these precise subtypes are is still a matter of debate. The most common approach is to divide color into hue, brightness, and saturation (Tkalčič and Tasič, 2003; Lotto and Purves, 2000; Long et al., 2006), but other frameworks exist. One prominent alternative includes brightness, lightness, colorfulness, chroma, and hue (Fairchild, 2013; Hilbert, 2005), while another posits a six-component system in which the hue, brightness, and saturation of both the object’s surface and its illumination are represented in experience (Tokunaga and Logvinenko, 2010; Davies, 2016).

Despite such variation, nearly all decomposition schemas include hue and brightness. *Hue* is the quality that color possesses that distinguishes red from green, blue from yellow, and so on, and is what is lacking when one sees something in just black and white. *Brightness* is something along the lines of the perceived intensity of light reflected from a surface. It is experienced in terms of *lightness*, and can either modify a hue (e.g., making blue appear as light blue) or appear on its own as black, white, or shades of gray. These two relatively uncontroversial components of color experience (i.e., hue and brightness) will be our focus here. Accordingly, we will take base experience to consist of the arrangement of *hues* and *brightnesses* that are found in visual experience.

We can now offer a final formulation of the idea of base experience:

Base experience: the arrangement of hues and brightnesses that are found in our conscious visual field.

Before moving on, it should be noted that the kind of color content studied here is only one among several types of conscious color content. Our focus is on lower-level sensory color content—specifically, hue and brightness (Kozuch, 2024, under review)—but visual experience plausibly also includes higher-level forms of color representation. One such form is *surface color*: a representation of an object’s color that remains relatively stable across changes in illumination (Russell, 1912; Peacocke, 1983; Hilbert, 2005; Davies, 2016). A familiar illustration is provided by white sand at the beach, which may phenomenologically look orange or blue at sunset due to reflected light from the sky or water, while nonetheless appearing to remain white in its actual color. There might also be higher-level forms of conscious color representation that are object- or feature-based, in such a way that it is unclear whether it is represented as being located in any specific region of the visual field. One candidate here would be *categorial* color representations (Kriegel, 2007; Kozuch, 2024), in which an object is represented as belonging to a color category—for example, representing a banana as yellow.

While a complete science of the NCC might ultimately need to identify the neural bases of each of these types of color representation (Kozuch, under review), the present framework is deliberately restricted to the lowest-level form of color content, *sensory color*, understood as hue and brightness. This restriction is methodologically motivated: sensory color appears especially well suited to a content-matching approach aimed at identifying its neural basis.

### 2.2 Outstanding questions concerning base experience

Having introduced the idea of base experience, we now consider three questions concerning its nature. While they are questions of interest in their own right, they are also ones that we can remain neutral about when using the concept of base experience as a methodological foundation for NCC research.

First, there is the question of whether, and how often, the (sensory) colors involved in base experience are represented as belonging to surfaces—for instance, whether the yellow seen when looking at a banana is represented as part of the banana’s surface. Introspectively, this often seems to be the case, but the methodology developed below does not depend on accepting that view.

Second, we might ask whether these colors (or the surfaces they belong to) are experienced as being located at specific distances from the viewer. While this appears to be frequently true, particularly in foveal or parafoveal vision, the region- and color-matching strategies that we propose below can remain agnostic on this point (but more discussion of this appears in Section 3).

Third, there is the question of how expansive base experience is, e.g., whether it encompasses all or a large portion of the visual field, or only a more limited portion. In Section 5.1, we offer reasons to favor the introspectively plausible view that—at least in the case of brightness—it is expansive, stretching more or less from one side of the visual field to the other (e.g., Wolfe, 1999; Kozuch, 2024). But even if base experience was more often confined to narrower regions, such as the fovea (cf. Cohen et al., 2020), our method still remains applicable, in that it could be used to find the neural basis of this less expansive base experience.

Some readers may be unsure whether there *truly* is an aspect of experience picked out by the concept of base experience. This is not important for our purposes. We are not aiming to establish that there *really is*—whatever exactly that means—an aspect of visual experience that is picked out by the concept of base experience. Rather, all we are trying to establish is that (a) some aspect of visual experience *can* be described in the way we propose below, and (b) this way of describing visual experience may be used to create a means for identifying NCC. Next we lay more foundations for the methodology.

## 3 Using content-matching to locate the neural basis of base experience

### 3.1 Content-matching methods

The method proposed here for identifying the neural basis of base experience is a form of *content-matching* (Noë and Thompson, 2004; Kozuch, 2015; Kozuch and Kriegel, 2015). A content-matching method looks for the neural basis of conscious experience by identifying matches in content between conscious experience and neural systems. The motivation for using content matching comes from the following principle:

*Isomorphism Constraint*: neural system N is the basis of experience E only if N and E match in content.

This principle is not controversial. It entails only claims such as the following: if some neural system N is to count as a candidate for being the neural basis of an experience of redness, then N must represent redness. If N instead represents greenness, it cannot be the neural basis of an experience of redness. We can see the motivation for the Isomorphism Constraint by considering apparently absurd scenarios in which an experience and its neural basis fail to match in content—for example, if an experience of red were somehow constituted by activity in a neural system in which the only color being represented was green.

The Isomorphism Constraint in place, we can now consider how it can be used to evaluate NCC hypotheses. Suppose a theory T claims that neural system N is the basis of experience E. Then, given the Isomorphism Constraint, T predicts that the content of N will match the content of E. For example, if T claims that neural system N underlies an experience of redness in central vision, then T predicts that N represents redness as being located in central vision. If this prediction is borne out, T passes this test, and N remains a viable candidate for the neural basis of E. If, however, N represents greenness in central vision—or represents no color there at all—then this disconfirms T, since N cannot be the neural basis of E.

A real-world instance of a content-matching method can be found in the now-ancient Logothetis experiments on binocular rivalry (Logothetis and Schall, 1989; Leopold and Logothetis, 1996; Sheinberg and Logothetis, 1997). These studies purported to show that content in the majority (~80%) of V1 neurons remained stable, representing the images from both eyes, while content in the vast majority (~90%) of IT neurons fluctuated, alternating between representing the image from one eye and then the other. In this case, the content of visual experience appears to mismatch the content of V1 but match the content of IT. As a result, the hypothesis that V1 is an NCC is disconfirmed, whereas the hypothesis that IT is an NCC remains viable (cf. Chalmers, 2000). Numerous other studies have employed a content-matching method in NCC research (e.g., Zeki, 1983; Tong et al., 1998; He and MacLeod, 2001; Brascamp et al., 2018; Panagiotaropoulos, 2012).

Now is a good time to say what we mean by the term *neural system*. We use the term broadly. A *neural system* might consist of a single anatomical region (e.g., V4), a subset of a region (e.g., deep layers of V2), or a functionally defined population distributed across multiple regions (e.g., more than one of the color-sensitive regions found in the ventral stream). For instance, if we relate this to the binocular rivalry example discussed above, these data are in line with the NCC being a population of neurons distributed across the visual hierarchy, with a larger contribution from higher-order regions like IT than lower-order regions like V1. We assume that what matters is not the anatomical unity of the system, but its capacity to carry representational content, i.e., to attribute specific visual features (such as color) to specific parts of the visual field. Accordingly, our method allows for both localized and distributed candidate NCCs, including hybrids such as, e.g., neural populations located in deep layers of multiple, contiguous ventral stream areas.

### 3.2 The requirements for content-matching

If we are to use a content-matching method to test the hypothesis that activity in neural system N forms the neural basis of some experience E, we need ways to represent the content of both N and E. Crucial here is identifying a common currency in which the contents of the two can be expressed, so that they can be meaningfully compared. In the case of base experience and neural systems, this common currency can be specified in terms of (a) which colors are represented, and (b) where in the visual field each of these colors is represented.

More precisely, in the case of experiential and neural content, they can both be described using two variables:

1. Regions: Those places in the visual field where a color is being represented.
2. Color values: The hue and brightness that are represented at each region.

The content of a *base experience* can be described using these variables since it consists of the conscious representation of one or more colors, each of which is represented as being located in some particular part of the visual field (Clark, 2000, Ch. 5; Clark, 2004; Kozuch, 2024). Neural color content can also be described in terms of regions and color values: the color value a neural state represents (e.g., hue or brightness) is determined by which color stimulus elicits the neural activity (Brouwer and Heeger, 2009; Kim et al., 2020), and the region it represents is determined by where in the visual field the stimulus must appear to evoke that activity (Engel, 1997; Dumoulin and Wandell, 2008; Arcaro et al., 2009).

Now we’ll describe in more detail how we can use this common currency of regions and color values to fix the content of base experiences and neural systems.

## 4 Base experience specification

This section provides a method for representing the content of base experiences and neural systems, what we’ll call a *base experience specification* (BES). Here is an intuitive example of a BES: If one visually experiences a yellow surface in the left half of their visual field and a red surface in the right, then one’s BES consists of a representation of yellow throughout the left visual field and a representation of red throughout the right.

To give a more formal specification of a base experience, we can appeal to the two components of content (the “common currency”) introduced above, that of regions and color values. More precisely, a complete base experience specification of some visual experience E will do the following:

1. List each of the regions in which a color is represented.
2. Assign a color value to each of these regions.

**Figure 3:**
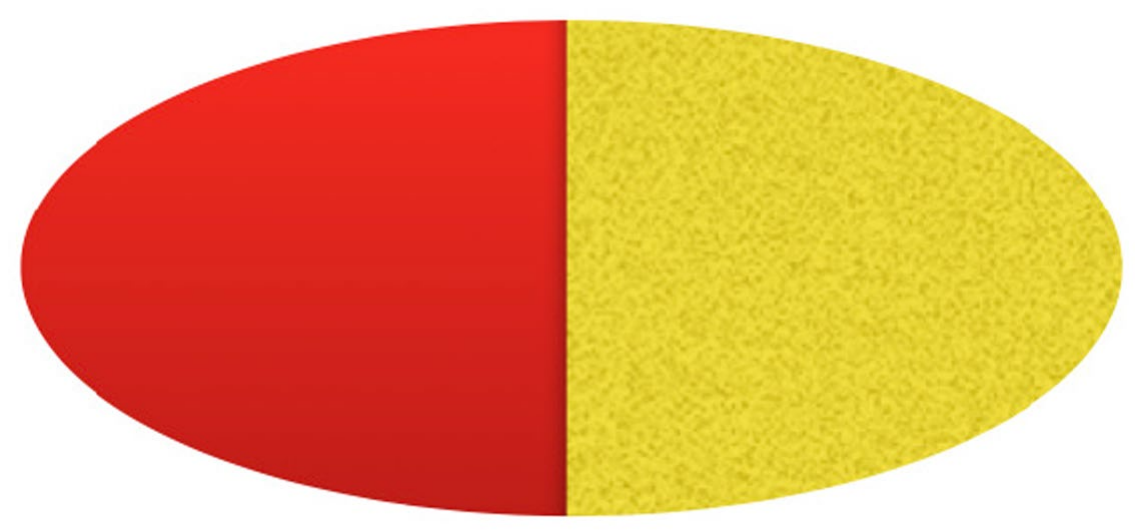
A simple demonstration of a base experience specification: See body for description.

Let us look at an example. Consider a scenario in which you experience your visual field as consisting of what is depicted in Figure 3.

A base experience specification of this would be as follows:

Region 1 (entire left half of the visual field): red.

Region 2 (entire right half of the visual field): yellow.

All we have looked at so far are simple examples, ones using qualitative terms to specify a base experience. A more quantitative approach follows. But first we further unpack the ideas of regions and color values.

### 4.1 Regions

When one experiences color, it is experienced as being located in some place in one’s conscious visual field. The place in the visual field in which it is represented as being located is what we have been calling its region. More formally:

Region: where in the visual field a base experience is representing a color.

Example: If redness is represented as being located in central vision, we can say that its region is central. If it is represented as being located right of center at eye level, then its region is right of center and at eye level.

We can more precisely specify a region using two variables:

- Location: distance and direction from the center of the visual field to the center of the region (given in degrees).
- Size: the angular size of the region (given in square degrees).

A more visually oriented demonstration of the concepts of location and size is found after the discussion of color values.

Note now that there is the option here to standardize the size that is used for each region. For example, one might take each region to be 0.1 square degrees. Whether or not to do this, and what size one should make to be the standard, can be dictated by one’s purposes.

For simplicity’s sake, we use standardized sizes in the examples given below. However, given that visual acuity becomes progressively less fine-grained the further away one gets from fixation (this being a function of the progressively increasing size of the receptive fields that neurons have as they represent more peripheral parts of the visual field), it might turn out that adopting a standardized size is not always desirable in practice. While this is an issue to which we will return below (Sect. 5.2), it is mostly set aside here in order to focus on more fully developing a framework for locating the neural correlates of base experience.

### 4.2 Color values

Each region will be assigned a hue and/or brightness, expressed qualitatively using terms such as “red,” “blue,” “high brightness,” or “low brightness.” For simplicity, we use qualitative terms in the example below. However, quantitative measures (e.g., specifying values in CIELAB space) could also be employed to enable more precise content matching between neural systems and experiences.

Having discussed regions and color values, we can go back to the concept of a complete base experience specification (BES).

A complete BES is a list of:

1. The location and size of each region in which a color is located.
2. The hue and brightness of each region.

### 4.3 Demonstration of a base experience specification (BES)

**Figure 4:**
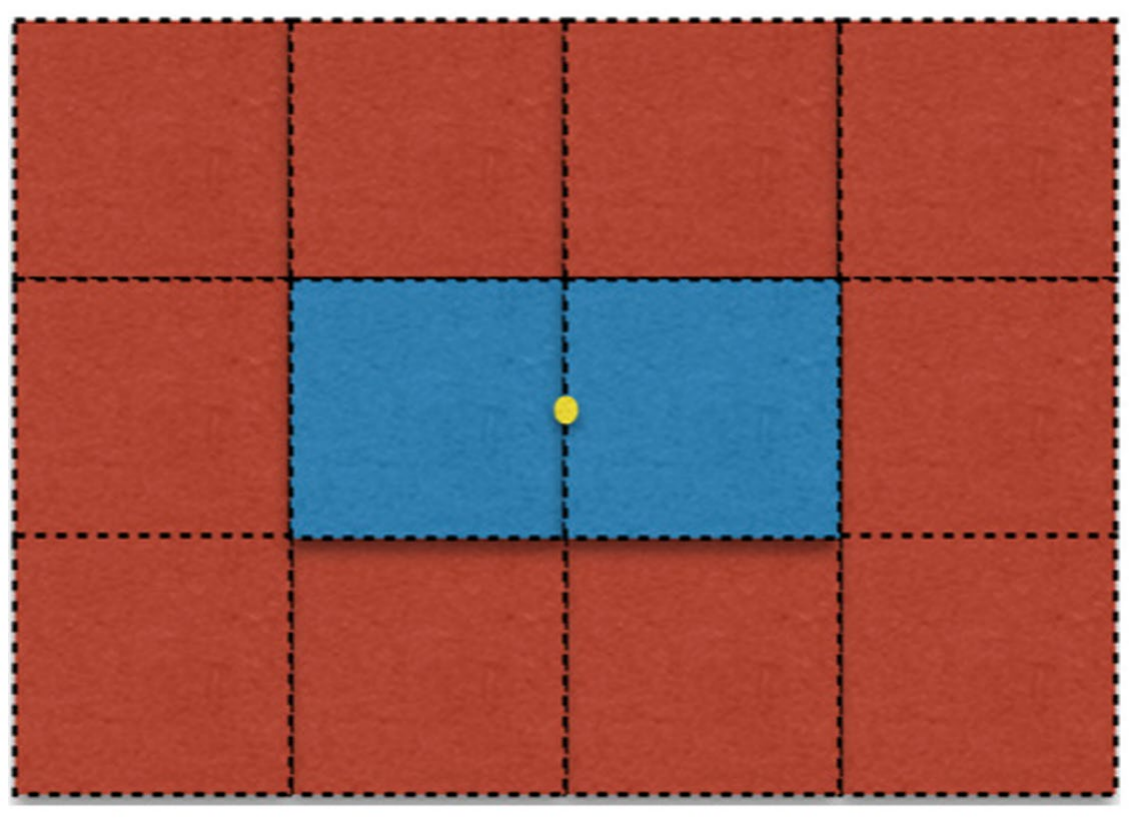
An example of a base experience, to be analyzed in Table 1.

Just below (Figure 4), you’ll find a visual experience whose base experience we are going to characterize. Here are some notes concerning it.

- Unlike the visual field, which is oval, the representation below is rectangular. This simplification helps convey the idea.
- Each square has an angular size of one square degree of the visual field.
- The dotted lines are not intended to be part of the visual experience, but are just there for demonstration. Same with the yellow dot, which marks the absolute center of the visual field (i.e., the point of fixation).

**Table 1:** An example of a more rigorous base experience specification (see body for description).

Region (degrees from center)	Size (square degrees)	Hue	Brightness
−1.5, 1	1	1	2
−0.5, 1	1	1	2
0.5, 1	1	1	2
1.5, 1	1	1	2
−1.5, 0	1	1	2
−0.5, 0	1	2	2
0.5, 0	1	2	2
1.5, 0	1	1	2
−1.5, −1	1	1	2
−0.5, −1	1	1	2
ETC.			

Next, we’ll give a base experience specification for the base experience just above. When doing so, we follow these conventions:

- *Region*: expressed as [A, B], where A indicates the longitudinal position of the center of the region relative to the visual field’s center, expressed in degrees, and B indicates the latitudinal position of the region relative to the visual field’s center, also expressed in degrees. (So, [0,0] is the absolute center of the visual field (the yellow dot)).
- *Hue*: 1 = red; 2 = blue.
- *Brightness*: 2 = mid-level of brightness.

The base experience specification for the above experience is seen in Table 1.

We’ve just presented a more formal way of specifying a base experience. Before further fleshing out, two issues should be addressed.

### 4.4 The representation of depth in base experience

Having demonstrated what a BES looks like, it is now a good time to return to an issue mentioned earlier; this is whether we should consider the colors that are represented in base experience to be represented as being some distance from the subject.

Here is the issue: As seen above, a base experience can be completely described by just saying what color is represented in each region of the conscious visual field. This means that the arrangement of colors that one is experiencing at any given moment can be accurately described using just two dimensions. However, the fact that we can describe base experience in this way does not mean that visual experience is two-dimensional: It might be the case that, in visual experience, it is not only that each color is represented as being located in a certain part of the visual field, but also that many or all of these colors (or the surfaces that they belong to) are represented as being at a certain distance from the subject. But whether or not this is actually the case need not be settled before the concept of base experience can be used in NCC research; all that matters is something that was shown above, which is that color experiences can be accurately described in terms of color values and regions.

### 4.5 The use of reports in constructing base experience specifications

A worry sometimes raised about report-based methods is that apparent mismatches between conscious experience and neural activity might reflect limitations of access or report, rather than a genuine mismatch between the content of a neural system and the content of conscious experience (Block, 1995, 2007; Kozuch, 2015). This worry can arise in more than one way. One possibility is that the mechanisms responsible for access or report represent visual content differently from the mechanisms underlying experience itself, for example by representing color at a coarser grain than experience does. In such cases, subjects’ reports might systematically underdescribe the fineness of experiential content, creating an apparent mismatch, one that reflects a limitation of report rather than a genuine difference between experiential content and neural content.

Concerns of this general sort are well motivated in paradigms that deliberately impose attentional demands or temporal constraints, such as inattentional blindness or visual masking (Simons and Chabris, 1999; Kozuch, 2014), as well as in cases where brain damage plausibly affects the retrieval or report of conscious content, such as hemispatial neglect or blindsight (Jacob and de Vignemont, 2010; Phillips, 2016). It is less clear that they apply with comparable force in the present context, where the contents used to fix base experience are drawn from ordinary perceptual judgments made under non-aberrant viewing conditions, for example, judgments about which colors, shapes, or textures are seen in specified regions of the visual field (e.g., Murray et al., 2006; Tyler, 2015; Cheng et al., 2023). Judgments of this kind are routinely relied upon throughout psychophysics and vision science and, absent independent reason for doubt, are typically treated as reliable. While report-based evidence must always be interpreted in light of general methodological limitations, this is not a special difficulty for the present framework, but a background feature of empirical research on conscious experience.

At the same time, although the construction of a BES inevitably begins with subjective reports, the framework allows these report-based results to be checked using no-report paradigms (Tsuchiya et al., 2015; Safavi et al., 2014), which can help to determine whether the content gathered under report conditions reflects the content of experience or of the demands of reporting. For example, if under report conditions base content can be decoded from a neural system in prefrontal cortex but not from areas in visual cortex, this might initially suggest that higher-order systems realize base experience; however, if under a no-report paradigm the same content is no longer decodable from prefrontal cortex, this casts doubt on the PFC as constituting base experience and suggests that the earlier failure to decode base content from visual cortex was methodological, rather than indicative of a genuine absence of base-experience content in visual cortex. As another example, base content might be decodable from both prefrontal and visual cortex under report conditions, but only from visual cortex under no-report conditions. In that case, the convergence across report and no-report paradigms would favor visual cortex as constituting base experience. Using comparisons of this kind, confidence in conclusions about the neural basis of base experience can be progressively strengthened.

## 5 Neural content specification

This section provides a method for representing the content of neural systems, one that complements the method just given for representing the content of base experiences. As noted earlier, neural content can be specified using the same variables used to specify a base experience.

This means that a neural content specification will describe the content of some neural system N (e.g., area V4) using the following two variables:

1. Regions: Those regions R1…n in the visual field where N is representing a color.
2. Color values: The hue and/or brightness that is represented at each region.

A complete neural content specification (NCS) will be one that (1) lists each of the regions that N is representing a color to be located, and (2) assigns a color value to each of these regions.

We now unpack the ideas of regions and color values as they are used in constructing an NCS.

### 5.1 Regions (neural content)

When a neural system represents a color, that color is typically represented as being located in a specific part of the visual field. This can be inferred from how a neural assembly that is representing color C usually responds only when C appears within a particular region of the visual field, namely, the region covered by its receptive field (Maeda et al., 2010). From this property of color-sensitive neurons, we can derive a form of content that complements the region-based content of base experience; we again use the term *region* to refer to it:

Region: where in the visual field a neural system is representing a color to be located.

Here are some qualitative examples: If a neural assembly within neural system N responds to the presentation of color C only if it is at fixation, we can say that the region at which that color is being represented is “central”; if it must be represented right-of-center but at eye level, we could say that the representation’s region is “right of center and at eye level.”

Like in the case of base experience, we can be much more precise in how we specify a region, doing so by using the two variables of location and size; these can be understood, mutatis mutandis, in the same way that it was defined in the section on specifying base experience. More precisely: the region that a neural assembly is representing some color C to be located can be specified using these two variables:

1. Location: distance and direction from the center of the visual field to the center of the region (given in degrees) that the neural system is representing the color to be located.
2. Size: the angular size of the region (given in square degrees) to which the neural system is attributing that color.

### 5.2 Color values (neural content)

Each neural assembly representing a region can also be assigned a color value according to what hue or brightness it responds to. We will again express these qualitatively, referring to hues using terms such as “red” and “blue,” and to brightnesses using terms such as “of high brightness” or “of low brightness.” As mentioned earlier, specifying neural color representations in quantitative measures like CIELAB space has the potential to enable precise quantitative matching with base experiences (Brouwer and Heeger, 2009), but we will keep the present descriptions simple and intuitive. As in psychophysics and systems neuroscience more generally, such color attributions are grounded in systematic response patterns across stimulus conditions and comparative similarity relations, rather than in any assumption that qualitative content can be read off from neural states in isolation.

### 5.3 Neural content specification

We can now express the concept of a complete neural content specification (NCS). This will be a list of the following:

1. The location and size of each region that the neural system attributes a color to.
2. The hue and/or brightness of each color that is attributed to each region.

For a more in-depth example of a neural content specification (NCS), we could use the same example that was used for the base experience specification (see Figure 4). The only difference would be that, in this case, we are attributing the content to a neural system rather than to an experience. Put another way, the same kind of specification shown in Table 1 above could be constructed on the basis of neural measurements, with each of the relevant variables being determined as follows:

- The regions would be determined according to what parts of the visual field the neural populations being measured showed responses to.
- The sizes would be determined by how large a portion of the visual field the neural populations showed responses to.
- The hue and/or brightness would be determined according to which specific hue and/or brightness the neural populations showed responses to.

Note: For ease of exposition, in the earlier example (Figure 4), the size of each region was standardized at one square degree. However, given that the receptive field sizes tend to increase as we move toward the periphery of the visual field, this is a significant simplifying assumption. But there is no reason to think that the system presented here could not be scaled up to account for these physiological facts.

### 5.4 Fixing spatial grain in neural systems

If we want to make *fine-grained* content matches between base experience and neural systems, we will need a way to at least roughly establish the spatial grain at which a neural system represents colors in the visual field. Put another way, the more effectively we can measure the spatial grain represented in a neural system, the more determinately we can evaluate whether it could constitute the neural basis of base experience (as seen in the “grain-matching” method discussed in 6.2). In the case of “the back-of-brain areas” in the visual cortex, the presence of retinotopic organization—whereby neighboring locations in the visual field are systematically mapped onto neighboring neurons or neural populations (Felleman and Van Essen, 1991; Engel, 1997; Wandell et al., 2007)—makes these areas especially well suited for application of the content-matching method proposed here. As will be seen in 6.2, retinotopy allows spatial grain to be fixed by appealing to receptive field size: if neurons responding to a given region of cortex are selective for a region of the visual field of a certain size, then that size can be taken—relative to the region of interest—to determine the spatial grain at which content is represented. For example, if neurons representing central vision respond to color within regions of approximately 0.1 square degrees, then the grain of representation in that area can be specified at that scale, with analogous determinations made for more eccentric regions.

Something of interest here is that receptive field sizes are very fine-grained in early visual areas but become progressively coarser along the visual hierarchy, reaching relatively large spatial extents in higher visual areas such as inferotemporal cortex. One task in determining the neural basis of base experience will be to locate those brain areas whose receptive fields are not too large or too small to match the grain of base experience. Given the comparatively coarse spatial representations found in higher visual areas such as IT (e.g., Wandell et al., 2007; Dumoulin and Wandell, 2008), and the apparently fine-grained spatial structure of base experience, especially in foveal regions (Carruthers, 2000, Ch. 11; Freeman and Simoncelli 2012), one might be tempted to conclude that areas like IT are poor candidates for constituting the neural basis of base experience. We set that issue aside in this article. Our present aim is simply to point out that retinotopy can provide a rich source of information for fixing the spatial grain of neural representations. Of course, this strategy might be less helpful in the case of neural systems that lack a clearly defined retinotopic organization (such as those in the PFC), a matter to which we return below.

It is important to stress that retinotopic organization is presented here only as one promising source of data for fixing base content in neural systems, not as a constraint on candidates for being the neural realizer of base experience. Retinotopy provides a particularly transparent way of fixing spatial grain and aligning neural representations with regions of the visual field, and we are fortunate that such organization is present throughout much of the visual cortex. However, nothing in the content-matching framework requires that base experience be realized only in retinotopically organized systems. What matters is not the particular neural format in which representations are instantiated, but whether the system in question represents colors and their spatial distribution at a grain that matches base experience.

Accordingly, there is no *a priori* reason to think that spatially structured color content could not be realized by non-retinotopic or more distributed neural representations (e.g., population-level codes), provided that those representations are capable of encoding both color and spatial layout with sufficient precision. At this stage, we therefore remain neutral about the precise neural format in which base experience is realized. The minimal requirement imposed by the present framework is simply that any neural system considered a candidate for realizing base experience be able to instantiate a representational structure from which the spatial layout and grain of base experience could, at least in principle, be recovered. How demanding this requirement is—and how it bears on different theories of consciousness—is taken up in Section 7.

### 5.5 The neural format of base experience

One might worry that, even if base experience provides an uncontroversial minimal description of visual phenomenology, it does not follow that there is a neural representation encoding base experience as such (i.e., in terms of regions and color values). Some aspects of that description might instead be emergent features of content realized in the neural substrate. If this is the case, one might further worry that the kind of content referenced in our specification of base experience would not be decodable from the underlying neural system. A related version of this concern applies to the dimensions used to characterize base experience themselves: even if hue and brightness are indispensable for describing visual phenomenology, it does not follow that they must be explicitly or primitively encoded at the neural level, rather than emerging from more fundamental relational or population-level coding schemes (Fleming and Shea, 2024; Lipman et al., 2025).

We agree that a neural system need not represent base experience in the same format used in our content-matching method. The spatial format we employ—regions defined in visual-field coordinates—is a methodologically advantageous way of characterizing the structure of base experience, not a requirement on the format of the neural code itself. What matters is not that neural representations mirror this descriptive schema, but that the content they carry be suitable for underwriting our phenomenology, that is, our spatially arranged experience of colors.

Accordingly, the content relevant to base experience should, in principle, be decodable from whatever neural system underlies it, even if that system employs a different representational format. For example, if a neural system represents a surface as blue, three feet away, and straight ahead, this content can be translated into the descriptive code employed by our methodology as there being a blue color located in central vision. Moreover, relative to that descriptive code, this translation is determinate: the only way in which this particular content can be translated into the framework used here is as a blue color located in central vision. Thus, although the brain may employ different coding schemes, there is no obstacle in principle to decoding neural content into terms relevant to base experience. What matters is not that the neural code mirrors the descriptive format used here, but rather that its content has the kind of structure that would be necessary for underlying base experience.

## 6 Implementing the content-matching method for base experience

As discussed above, if we want to determine whether some neural system is the neural correlate of base experience or not, we need a common currency in which the content of base experience and neural systems can be expressed. We found that a specification of the content of a base experience or neural system can be given in terms of what colors each represents, and where in the visual field each color is represented. What we do now is to look at how this system for assigning content to base experiences and neural systems can be parlayed into specific methods for determining the neural basis of base experience.

We’ll be looking at three methods: region-matching, grain-matching, and representation-matching.

To get a first-pass understanding as to what these three types are, consider a scenario in which we are trying to evaluate the hypothesis that neural system N is the neural correlate of base experience E.

Turning now to the three types of content-matching, each can be expressed as a question concerning what kind of content match might obtain between N and E:

1. Region match: Does N represent colors in each of the regions that E represents colors to be located, or does it fail to do so in the case of some of them?
2. Grain match: Does N represent the arrangement of colors at the same level of detail as it appears in base experience?
3. Color match: In each region that E represents a color to be located, does N represent the same color as E does?

In the case of any of our three methods, a match or mismatch between base experience and a neural system can be informative about whether the former is realized by the latter. We now examine each type of match in more detail.

### 6.1 Region match

If neural system N is the neural correlate of base experience E, then, for each region that E represents a color, N must also represent a color to be there. For example, if E included representations of those colors that were in most or all parts of the visual field that the visual system receives information from, and if N only represented those colors that were in foveal parts of the visual field, then the hypothesis that N was the neural basis of E is disconfirmed.

Using region-matching in the way just described requires having some idea as to how much of the conscious visual field is typically filled by the spatial arrangement of colors that one experiences. Put another way, the issue here concerns what “width” and “height” the visual field has in base experience. This could be roughly expressed by saying how many longitudinal and latitudinal degrees of the visual field are represented in visual experience. (We use the term “roughly” here since the part of the visual field that the eyes receive information from is oval in shape). Determining the width and height of base experience might not be trivial. Let us consider the issue.

When introspecting on visual experience, it appears as if the arrangement of colors that one experiences is relatively expansive, in that this arrangement appears (at least *appears*) to fill out most or all of the parts of the visual field from which our eyes are receiving information (this being about 40 degrees in latitude and 80 degrees in longitude) (Traquair, 1927). But it could turn out that our conscious visual field (i.e., our base experience) is much smaller than this. There is the possibility, for example, that the extent of base experience is limited to those parts of the visual field to which we are attending (if this were foveal vision, it would be roughly three degrees in both latitudinal and longitudinal directions). Such a view would perhaps be favored by theorists who believe that attention might be necessary for consciousness (Prinz, 2012; Cohen et al., 2016), since the amount of visual field that we are able to attend to at any one time might be limited.

There is, however, notable reason to think that base experience might tend toward being more expansive than this, coming from introspection. When we reflect on our visual experience, it *seems* as if a color is represented in each part of the visual field that we receive information from: for every region in view, it appears as if our experience represents the hue and/or brightness of whatever surface is located there. This is likely why many researchers endorse the idea that visual experience is informationally rich (Wolfe, 1999; Pollen, 2007; Chuard, 2007; Tononi et al., 2016; Haun et al., 2017; Kozuch, 2019, 2022, 2024, under review), with this belief probably being driven in part by the way in which base experience appears to be expansive and detailed. Even those who challenge the idea that visual experience has this kind of expansiveness agree that it at least *appears* to have it (e.g., Cohen et al., 2016; Blackmore et al., 1995; O’Regan, 1992; but see Jaynes, 2000).

However, while this introspective observation provides defeasible justification for thinking that base experience is expansive, it alone cannot be considered to provide strong reason to think it actually is, not without first considering the countervailing arguments and data that have been offered to challenge this view (e.g., Simons and Chabris, 1999; Knotts et al., 2018; Cohen et al., 2020). An in-depth look at this issue is beyond available space, but it is worth noting that a recent survey and analysis of these arguments and data suggest that they fail to undermine the belief that visual experience is “rich” in this way (Kozuch, 2024, esp. Sects. 2.3 and 3; see also, Kozuch, under review).

What matters for present purposes is that we need not wait for this debate to be resolved before we can employ the region-matching method that we have been considering. One way that the method can be used is by testing whether candidate neural systems can be region-matched to even the most minimal conception of base experience—i.e., one in which it is typically confined to the foveal region of the visual field—since if some neural system N fails to match even this minimal version, then this would disconfirm the hypothesis that N forms the neural basis of base experience. Additionally, if we have some reason to favor a more expansive view of base experience (e.g., Carruthers, 2000, Ch. 9; Kozuch, 2024), and a neural system N fails to region-match this more expansive conception, then—while this is no refutation of the hypothesis that activity in N constitutes base experience—it still counts as evidence against it.

### 6.2 Grain match

If a neural system is the neural correlate of base experience, its representations of color cannot be too fine- or coarse-grained: It cannot represent the spatial arrangement of colors at a level of detail that is less or more than what appears in visual experience. This means that, if some neural system N is to be the neural correlate of base experience E, then the size of the regions that it is attributing colors to in the visual field cannot be larger or smaller than those regions of color that are in base experience.

For example, if the colors that are in central vision are represented with a resolution of 0.1 square degrees, but some neural system N represents those regions at a finer grain (e.g., 0.05 square degrees) or a coarser grain (e.g., 0.5 square degrees), then N cannot be a candidate for being the neural basis of base experience.

Once we have determined the grain of base experience E and of some neural system N, then the hypothesis that N is the neural basis of base experience could be disconfirmed by the finding that the two failed to match in their grain. The closest example of this kind of test we know of is by Freeman and Simoncelli (2012), who used grayscale metamers. These researchers did not directly compare the grain of neural systems and experiences, but rather the slope with which the grain decreases with eccentricity in both experience and in different brain areas. We believe it would be valuable to extend this approach to color images, looking for grain matches directly rather than indirectly.

For us to carry out grain-matching, we’ll need methods for determining (a) at what grain visual experience represents colors, and (b) at what grain each potential neural correlate for base experience represents colors. Fleshing these methods out is a task going beyond available space. We can, however, provide suggestions as to what this process might look like.

In the case of base experience, one promising way to establish the grain of it is to use metamers, visual stimuli that differ in their properties but are indistinguishable in conscious perception. In vision science, metamers have most widely been used in color perception, where it has been shown that color stimuli that differ greatly in their spectral power distribution can be perceived as being of an identical color. More recently, what we might call grain metamers have been used, versions of the same image that vary in their level of detail but which are perceived as being identical (Anstis, 1998; Freeman and Simoncelli, 2012; Broderick et al., 2023). In the case of grain-matching, the idea here would be that one can find the grain of base experience by gradually reducing the size of the regions of color until further decreases are all metameric, using the point at which the images crossover into indistinguishability to identify the grain of base experience.

In the case of neural systems, one natural way to fix the grain of a color-representing neural system—at least in many visual areas—is by determining the size of the receptive fields of its neurons or neural populations (Felleman and Van Essen, 1991; Engel, 1997; Dumoulin and Wandell, 2008; Arcaro et al., 2009; Freeman and Simoncelli, 2012). In many visually responsive brain areas, this task is facilitated by retinotopic organization, whereby neighboring locations in the visual field are systematically mapped onto neighboring neurons or neural populations. Retinotopy allows spatial grain to be fixed by appealing to receptive field size: if neurons responding to a given region of cortex are selective for a region of the visual field of a certain size, then that size can be taken—relative to the region of interest—to determine the spatial grain at which content is consciously represented. For example, if neurons representing central vision respond to color within regions of approximately 0.1 square degrees, then the grain of representation in that area can be specified at that scale, with analogous determinations made for more eccentric regions.

At the same time, the point made earlier about retinotopy should be kept in view. Retinotopy is useful here because it provides a relatively direct way of fixing spatial grain and aligning neural representations with regions of the visual field, especially in visual cortex, the part of the brain most likely to contain the content NCC for low-level sensory experience (see Kozuch, 2024 and Section 7.3). But retinotopy here remains only a methodological resource, not a constraint on how base experience must be neurally realized. A non-retinotopic or distributed system could still qualify, provided that its activity encodes color and spatial layout with enough precision for the spatial structure and grain of base experience to be recovered from it. The implications of this requirement for competing theories of consciousness will be considered in Section 7.

### 6.3 Color match

The third method looks to see whether there is a match between what color base experience and a neural system are representing to be in some region or regions of the visual field. To give a simple example: if base experience E is representing there to be redness at fixation, and neural system N is representing redness at fixation, then N can be considered to remain a viable candidate for being the neural basis of E; but if N were to be representing some other color (e.g., greenness), then the hypothesis that N is the neural basis of E is disconfirmed.

Since it is true of both base experience specifications (BES) and neural content specifications (NCS) that they have the potential to be very precise in how they represent the content of a base experience or neural system (since very small-sized regions could be used in their specifications), this means that very precise forms of content-matching can be carried out. This gives us the potential to uncover very fine-grained similarities or differences between the content of base experiences and neural systems.

In the case of color matching, *size illusions* might be used to good effect. If certain interpretations of size illusions such as the Titchener or Müller-Lyer illusion are correct (Ross, 2003), then such illusions are illusions of *angular size*: They cause the illusory object to appear to take up more of the visual field than it actually does. This entails that base experience represents certain regions of the visual field as having the color of the illusory object rather than that of the background. In that case, only those neural systems that represent the object’s color as being located in those regions are candidates for being the neural basis of that base experience (cf. Kozuch, 2025).

To see an example of this, let us consider a scenario in which the Titchener illusion does include an illusion of angular size, which means that the central disk surrounded by the ring of small circles would phenomenologically appear to occupy more of the visual field than it actually does. Put another way, because of the disk’s “inflated size,” there would be a number of coordinates C1…n that are located just outside the border between the disk (i.e., the disk’s *actual* angular size) that would, in the base experience specification (BES), not be filled by the color of the background (i.e., the color that is actually located in that part of the visual field) but rather by the color of the disk (Kozuch, 2025).

This inconsistency between what the BES contains in that part of the visual field and what is actually located there can be used to test the hypothesis that some neural system N is the neural basis of the illusory experience E (cf. Murray et al., 2006). To wit, if one determines what the neural content specification (NCS) is for N during E, and if it is found that coordinates C1…n are filled by the color of the disk, this would be confirmation of the hypothesis the N is the basis of E; if it is found that these coordinates are filled by the color of the background, this disconfirms the hypothesis.

Color-matching can be employed in a variety of ways to test hypotheses about the neural basis of base experience. Any time a match or mismatch can be identified between the coordinates composing a base experience space (BES) and those composing a neural candidate system (NCS), this can serve as evidence for or against a given neural system being the basis of the experience under consideration.

Several examples of color-matching can already be found in the literature. One comes from a study by Cheng et al. (2023), which found that enlarged illusory colored surfaces produced by a spreading color illusion could be readily detected in higher visual areas (such as LOC, PPA, and FFA), but not in earlier areas like V1 or V2. (In contrast, illusory oriented lines and small illusory colored dots could be detected even in these early areas). Given that these data suggest the spreading color is not represented in V1 or V2, there appears to be a lack of content match between these areas and the visual experience; this counts as evidence against either area serving as the neural basis of that experience. In a similar vein, Kok and de Lange (2014) have shown that illusory shapes lead to increased activity in parts of V1 with receptive fields on the illusory surface, a result suggesting that V1 remains a viable candidate for supporting the conscious representation of that object’s shape, and possibly also its color (hue and/or brightness). Future research should aim to develop other ways to reveal color matches and mismatches between base experience and neural representations.

## 7 Content-matching and theories of consciousness

### 7.1 Methodological constraints on content matching

The content-matching method developed in this article provides a framework for assessing theories of consciousness by constraining which neural systems could plausibly realize base experience. If a theory predicts that base experience is represented in a particular neural system, but no content-matching representation can be decoded there, this would count as disconfirming evidence. Such results, however, must be interpreted cautiously. Base content may be harder to decode in later stages of the visual processing stream—such as those in prefrontal areas—due to the weakening of orderly retinotopic organization. As retinotopy degrades, so too does the spatial regularity that many decoding methods might otherwise exploit (Freeman and Simoncelli, 2012). A failure to decode fine-grained content in such areas, then, cannot be treated as unqualified evidence against a theory.

This caution extends even within the sensory hierarchy itself. Limitations in current neuroimaging methods constrain content matching not only in higher-order areas, but also in higher-level sensory regions. Neurons in areas such as IT cortex have substantially larger receptive fields than those in lower-order areas like V1, and therefore represent visual content at a coarser spatial grain (Hochstein and Ahissar, 2002; Dumoulin and Wandell, 2008). At the same time, the total tissue volume of these higher-order areas is much smaller (Wandell et al., 2007). Thus, if both IT and V1 represent the full visual field, 1 mm^3^ of tissue of IT cortex will necessarily represent a greater proportion of the visual field than a corresponding 1 mm^3^ of V1, even if neurons’ receptive field size were identical. Therefore, if we sample both regions with the same spatial resolution, as we do with fMRI, the difference in grain between higher-order and lower-order areas will be exaggerated.

Importantly, such biases can in principle be taken into account in neuroimaging analyses, and they do not arise in the same way for single-unit or population-level recordings in animal models. We are therefore cautiously optimistic that, despite its limitations, high-resolution fMRI can be used to identify meaningful matches between the content of base experience and neural systems. One reason for this optimism is that the population receptive field properties estimated from fMRI voxels align well with those measured using invasive electrophysiology, both across eccentricity and cortical depth (Fracasso et al., 2016). Another is that color representations can be reconstructed from multivoxel activity patterns (Brouwer and Heeger, 2009). Together, these results suggest that the core elements required for estimating a neural content specification (NCS) are already in place.

A separate question concerns how easily content matching can be carried out in brain areas lacking clear retinotopic organization. The PFC is the central case here. Although some studies report retinotopic maps in prefrontal regions (e.g., Jerde et al., 2012; Jerde and Curtis, 2013), any retinotopy present seems less systematic and less readily discernible than in visual cortex. Even so, if a neural system in the PFC were to underlie base experience, it would still have to represent the spatially structured color content found in visual experience, albeit in a different representational format. The relevant question, then, is the decodability of such content.

Although fine-grained decoding of base experience–type spatial color content—at the spatial grain characteristic of sensory color experience—has not yet been established for non-retinotopic areas, there is strong evidence that visual content can be decoded from prefrontal activity more generally. Visual information has been decoded from prefrontal neurons in nonhuman primates (Panagiotaropoulos, 2012; Bellet, 2022; Kapoor, 2022), as well as from frontoparietal regions using intracranial EEG in humans (Vishne et al., 2023). More importantly, the absence of classical retinotopic maps in prefrontal cortex has not prevented the decoding of spatial information in general. For example, phase-encoded fMRI has been used to identify orderly, contralateral maps of visual space in posterior dorsolateral prefrontal cortex (Hagler and Sereno, 2006). Other evidence comes from single-unit recordings in nonhuman primates, which show that prefrontal neurons are characterized by spatially selective “memory fields,” tuned to particular regions of visual space during delay periods of working-memory tasks (Funahashi et al., 1989; Rainer et al., 1998). At the population level, neurophysiological studies show that spatial information in prefrontal cortex is decodable despite the absence of large-scale retinotopic maps, with spatial structure emerging from local microcircuit organization rather than surface-level topography (Constantinidis and Qi, 2018). Spatial information has also been decoded in cases involving dynamically changing spatial properties, such as those associated with saccadic eye movements (Melcher and Morrone, 2015).

Taken together, these findings suggest that although the absence of retinotopy increases methodological difficulty, it does not in principle preclude the application of the content-matching method in such areas, provided that the neural system in question genuinely carries the spatially structured color content characteristic of base experience.

### 7.2 Base experience and theories of consciousness

With the above general methodological considerations in view, we can now ask what the results of a scientific investigation of base experience might tell us about competing theories of consciousness. The discussion begins with a taxonomy of theories of conscious neural *content*—that is, theories concerning which neural systems constitute the content of experience, as opposed to merely enabling or supporting it (for discussion: Raccah et al., 2021; Kozuch, 2024). According to *Sensory Theories* of conscious neural content (e.g., Lamme, 2006, 2010; Kozuch, 2023), the content of visual consciousness (and therefore base experience) is constituted by activity within the visual cortex. By contrast, *Cognitive Theories* of conscious neural content (e.g., Mashour et al., 2020; Brown et al., 2019) hold that the content of consciousness is constituted by activity outside of the visual cortex, often in the PFC or a broader frontoparietal network.

Among proponents of Sensory Theory, a further distinction can be drawn. *Pure* Sensory Theorists (e.g., Lamme, 2006; Kozuch, 2023; He, 2023) hold that activity of the appropriate kind in visual cortex is sufficient for constituting visual consciousness, though additional background conditions may need to obtain (see Block, 2009; Kozuch, 2023). *Enabling* Sensory Theorists (e.g., Lau, 2019; Fleming, 2020; Michel, 2025) agree that visual cortical activity can constitute the content of consciousness, but maintain that such content becomes conscious only if it is enabled by activity elsewhere in the brain, typically in prefrontal or frontoparietal regions.

In the case of (either kind of) Sensory Theory, the prediction generated by the present framework is relatively straightforward. If conscious visual content is constituted by activity in sensory areas, then a sufficiently fine-grained match between the spatial structure of visual experience and activity in visual cortex should, in principle, be recoverable. Accordingly, a systematic failure to identify such matches across sensory areas would count as substantive disconfirming evidence against the theory.

In the case of Cognitive Theory, matters are less straightforward. Cognitive theories typically locate the constitutive basis of conscious content in higher-order areas such as PFC, yet these are precisely the regions in which fine-grained content matching might be less straightforward. PFC often lacks stable retinotopic organization (Wandell et al., 2007; but see Silver and Kastner, 2009), and neural representations there often exhibit mixed selectivity and substantial trial-to-trial variability, even when stimulus conditions are held fixed (Rigotti et al., 2013; Fusi et al., 2016). When combined with the spatial resolution limits of current neuroimaging techniques, these features mean that failures to recover fine-grained spatial color content from PFC may, at present, carry less evidential weight against Cognitive Theory than analogous failures would carry against Sensory Theory.

This asymmetry, however, should not be taken to impose severe limits on the informativeness of the present methodology. A failure to recover fine-grained spatial color content from the PFC need not reflect limitations of current decoding techniques. It could instead indicate a mismatch between the structure of base experience and the kinds of representations typically attributed to prefrontal systems—representations that are often characterized as encoding a limited amount of task-relevant information in an abstract format (Friedman and Robbins, 2022) and that do not preserve the fine-grained spatial and color detail characteristic of base experience.

This interpretation, of course, is only one among several live possibilities. Nonetheless, it connects with a broader set of independent considerations that have been taken to place pressure on PFC-based cognitive theories of consciousness. In particular, the apparent richness and fine-grained spatial structure of visual experience (e.g., Carruthers, 2000, chs. 9, 11; Chuard, 2007; Kozuch, 2024), together with well-established limits on higher-order PFC functions such as working memory and attention (e.g., Brady and Tenenbaum, 2013; Scimeca and Franconeri, 2015), seem to make it antecedently less likely that prefrontal systems can accommodate base experience (Kozuch, 2024). On this view—if it turned out correct—difficulties in decoding base-experience-level content from PFC reflect a genuine representational mismatch rather than the current limits of decoding technology. Determining whether this is in fact the right explanation remains a matter for future research.

## 8 Conclusion

In this piece, we have pointed out a certain aspect of visual experience, base experience, and outlined a method that can be used to find its neural basis. We provided a way of specifying the content of base experience and neural systems, one that is precise enough to allow for multiple and fine-grained ways of looking for matches or mismatches between the content of visual experience and neural systems. While there are some details that were left unspecified in regard to how exactly the system is to be implemented, it is our hope that the general framework presented here looks promising enough to motivate filling them in, so that the methods outlined above might be put into practice.

## Data Availability

The original contributions presented in the study are included in the article, further inquiries can be directed to the corresponding author.
